# Mexican consensus about surgical treatment in early-stage cervicouterine cancer

**DOI:** 10.3389/fonc.2024.1385887

**Published:** 2024-06-19

**Authors:** Cindy A. Nájera-Muñoz, Raúl Hernández-Romero, David Isla-Ortiz, Rosa A. Salcedo-Hernández

**Affiliations:** ^1^ Gynecology Oncology Division, High Speciality Hospital Christus Muguerza, Monterrey, Nuevo Leon, Mexico; ^2^ Gynecology Oncology Division, National Cancer Institute (INCan) of Mexico, Mexico City, Mexico

**Keywords:** cervical cancer, surgical treatment, cervicouterine cancer, hysterectomy, sentinel lymph node

## Abstract

**Introduction:**

Cervical cancer is a public health problem in our country and worldwide. Less than 25% of cases are diagnosed in the early stages, where survival is more remarkable than 90% at five years. Here, we review surgical treatment in the early stages of cervical cancer.

**Methodology:**

A literature review was carried out in the MEDLINE database. The search was mainly limited to the English language, with priority given to systematic reviews with or without meta-analysis and randomized studies. However, only retrospective or observational evidence was found for some topics.

**Results:**

The standard treatment for early-stage cervical cancer is hysterectomy, and its radical nature will depend on the tumor size, lymphovascular permeation, and tumor-specific prognostic factors. Furthermore, the type of surgery (hysterectomy or trachelectomy) will rely on the patient’s desire to preserve fertility. Nodal evaluation is indicated as part of the treatment from stage IAI with PLV. However, the sentinel lymph node is more relevant in the treatment. The incidental finding of cervical cancer after a hysterectomy requires a multidisciplinary evaluation to determine the therapeutic approach. Less radical surgery has been described as oncologically safe in low-risk groups.

**Conclusion:**

Surgical treatment in its early stages has evolved in recent decades, making it more individualized and seeking less morbidity in patients without compromising their survival.

## Introduction

Cervical cancer (CC) represents a significant public health issue, ranking as the fourth most common neoplasm in women worldwide in terms of both incidence and mortality. Eighty-five percent of patients with this diagnosis reside in low-income countries, and despite the existence of effective primary and secondary prevention measures, diagnosis often occurs at advanced stages (1).

In the early stages, the standard treatment involves hysterectomy and node evaluation (bilateral pelvic lymphadenectomy). The extent of hysterectomy varies depending on the disease’s conditions and the patient’s desires regarding fertility preservation. However, in recent years, there has been a shift towards less radical surgery in low-risk patients and the incorporation of sentinel lymph node procedures. However, it is important to mention the necessity of conducting an appropriate diagnostic approach and staging the patient to determine the treatment. In 2018, FIGO updated this staging, where clinical, radiological, and pathological findings (if available) will be part of the criteria to determine the clinical stage of the disease. Imaging studies (ultrasound, computed tomography imaging, magnetic resonance imaging, and PET-CT) used will depend on their availability and access; however, it is important to utilize all available tools to determine the clinical stage of the patient (2).

A thorough review of the literature and critical evaluation of it was conducted concerning the surgical treatment of early-stage cervical cancer. The search was restricted to evidence in English and the MEDLINE database, with priority given to controlled, randomized studies and systematic reviews with meta-analyses. However, all available evidence was examined in cases where such studies were lacking on certain topics.

## Methods

A literature review was conducted by 4 individuals in the MEDLINE database using PUBMED and OVID search engines. The search was primarily limited to English, Spanish, and Italian language literature, with priority given to systematic reviews with or without meta-analysis and randomized studies. The identified studies were screened by the authors based on the abstract, and the chosen articles were retrieved for full reading and analysis. The quality of articles included in this review was assessed using GRADE and risk of bias tools. In the initial search including the mesh terms “Uterine cervical neoplasms” and “surgical procedures, operative”, the search retrieved 28,263 articles, those in other languages different to English, Spanish and Italian were excluding, remaining 23,864 cases. From this, the articles corresponding to randomized clinical trials and systematic reviews were considered, remaining 380; then, after reviewing the abstracts of the articles, manuscripts about advanced stages and rare subtypes were excluded, remaining 62 references. Finally, after reading the full papers, 14 were excluded because they were from the same group of investigators (duplicated information or follow-up in different times of the same cohort, etc.), the authors included patients with advanced disease, or the title and abstract were misleading about the treatment or the stage. In total, 48 articles were included.

## Surgical treatment

### Hysterectomy

Surgery is the cornerstone of treatment for patients with cervical cancer (CC) in the early clinical stage (for this paper the FIGO staging 2018 is used). The radical nature of the surgery, as per the Querleu-Morrow classification, is determined based on tumor size and the presence of lymphvascular permeation (LVP) in tumors with microinvasion. The choice between hysterectomy or trachelectomy is influenced by the patient’s desire to preserve fertility. In cases where surgery is contraindicated, radiotherapy becomes the treatment of choice, yielding similar oncological outcomes but with a different toxicity profile (3).

Historically, the Piver classification was utilized to describe the radicality of hysterectomy. However, it had limitations regarding anatomical description. Therefore, Querleu-Morrow introduced a new classification in 2008 based on anatomical references and avascular spaces, leading to improved standardization of procedures globally (4).

### Clinical Stage IA1

Cervical cancer in clinical stage IA1 is classified as microinvasive (stromal invasion < 3 mm). The radical nature of hysterectomy is determined by the presence or absence of LVP, as it correlates with lymph node involvement and recurrence (5). Patients without LVP have a lymph node involvement probability of less than 1%, thus indicating a simple hysterectomy (type A) without lymph node evaluation (6). For patients with LVP, the risk of lymph node involvement increases to 3 to 5%. Therefore, a type B hysterectomy with lymph node evaluation is recommended (5).

### Clinical Stage IA2

In the IA2 clinical stage, the probability of lymph node involvement ranges from 5% to 13% (7). It is recommended to perform a type B hysterectomy with lymph node evaluation (8).

### Stages IB1, IB2, and IIA1

For patients with stages IB1, IB2, or selected IIA1 clinical stages, the recommended treatment is a type C1 hysterectomy with lymph node evaluation (bilateral pelvic lymphadenectomy BLP, or node sentinel GC) (8). The likelihood of ovarian metastasis is 0.9% in squamous cell carcinoma, permitting consideration for ovarian preservation in premenopausal patients. However, in adenocarcinoma, the probability of ovarian metastasis increases to 5%, warranting bilateral salpingo-oophorectomy (BSO) in some cases (9).

**Highlights:**

1. Standard treatment for stage IA1 CC without LVP is a type A or cone hysterectomy, primarily due to fertility preservation, without lymph node evaluation.
2. Treatment for stage IA1 with PLV and IA2 involves a type B radical hysterectomy and bilateral pelvic lymphadenectomy (BPL) + pelvic para-aortic lymphadenectomy (PPAL).
3. For stages IB1, IB2, and selected IIA1 CC, radical hysterectomy type C1 and BPL + PPAL are recommended.
4. Ovarian preservation may be considered in premenopausal patients with cervical cancer and squamous cell histology.

### Fertility preservation

In patients with stage IA1 CC without PLV who wish to preserve fertility, performing a cone with negative margins without lymph node evaluation is recommended (10). For patients with stages IA2 and IB1, less than 2 cm without LVP, stromal involvement of less than 10 mm, squamous cell carcinoma histology, and adenocarcinoma, a simple cone or trachelectomy with lymph node evaluation could be considered, with a recurrence risk of less than 4% (10). However, if there is PLV, radical trachelectomy with lymph node evaluation is necessary (11). The use of neoadjuvant chemotherapy for patients with early-stage CC with tumors >2 cm who wish to preserve fertility and seek to reduce tumor size is under investigation. Currently, only retrospective studies have shown promising results, both oncologically and obstetrically (12). Currently, the CONTESSA study is ongoing, which is a prospective study with a primary objective of evaluate the feasibility of fertility preservation after neoadjuvant chemotherapy (platinum/paclitaxel) in patients with stage IB2 (2–4cm) CC, however; this is far from an efficacy study. Because there are no randomized studies that demonstrate the oncological safety of neoadjuvant chemotherapy in this setting, this type of treatment cannot be considered standard of care (13).

**Highlights:**

- Cone is indicated in stage IA1 without LVP.
- Cone or simple trachelectomy may be considered in stages IA2 and IB1 with low-risk factors.
- Radical trachelectomy is indicated in stages IA2 and IB1 with LVP.
- Neoadjuvant chemotherapy for tumor size reduction in patients wishing to preserve fertility is not recommended as a standard treatment.

### Surgical approach

Since the first description of laparoscopic radical hysterectomy by Nezhat et al. in 1992, this approach has demonstrated feasibility and safety (14). Retrospective studies on laparoscopic approaches in cervical cancer treatment consistently showed benefits of minimally invasive surgery with similar oncological outcomes to open surgery (15–17).

In 2018, the multicenter randomized phase III LACC trial (18) was published, demonstrating that minimally invasive radical hysterectomy was associated with lower rates of disease-free survival and overall survival compared to open abdominal radical hysterectomy in early-stage FIGO 2018 stage IA1 cervical cancer with LVP to IB2. Following this study, treatment guidelines revised their recommendations, favoring open surgery as the approach of choice for early cervical cancer treatment, with minimally invasive approaches reserved for IA1 cervical cancer without LVP and research protocols for others early stages (19–21).

After this change in clinical practice, researchers have explored if there is a subset of patients who could benefit from minimally invasive approaches or if specific maneuvers to avoid tumor manipulation could mitigate the increased risk of recurrence and mortality associated with minimally invasive surgery. However, all published studies are large retrospective cohorts with inherent selection biases, necessitating caution in interpreting their recommendations (22, 23).

**Highlights:**

1. Standard surgical management in early-stage cervical cancer FIGO 2018: 1A1 with lymphovascular invasion to IB2 is open surgery.
2. Minimally invasive surgery (laparoscopy and robotics) is not recommended for early-stage invasive cervical cancer.
3. Minimally invasive procedures should only be performed within research protocols for cervical cancer.

### Sentinel lymph node

In patients with apparent early-stage CC, nodal status stands as the most crucial prognostic factor (24, 25). The standard treatment involves hysterectomy or radical trachelectomy with pelvic lymphadenectomy. However, lymph node metastases are detectable in only approximately 15–20% of cases treated with surgery (26), this implies that 80–85% of patients could undergo unnecessary surgical overtreatment, exposing them to procedural morbidity such as lymphoceles and lymphedema (27, 28). The sentinel lymph node (SLN) serves as the initial nodal basin receiving tumor lymphatic drainage, indicating the status of other lymph nodes in the region. This concept allows for avoiding complete lymphadenectomy (29) and this technique (SLN) has been regularly and safely employed in gynecological malignancies like vulvar and endometrial cancer. Presently, active prospective protocols (SENTIX, SENTICOL III, PHENIX) aim to establish the oncological safety of SLN use without pelvic lymphadenectomies and we wait the results in coming years (30–32).

International guidelines, such as ESGO/ESTRO/ESP, recommend SLN as the initial step in surgery, subjecting it to intraoperative evaluation to determine further surgical management, including completing pelvic lymphadenectomy or referral for chemoradiotherapy (33). NCCN recommends SLN in selected stage I cases (<2 cm) and remove the suspicious or enlarged lymph nodes; also, advises lymphadenectomy if SLN mapping fail. FIGO suggests SLN only for stages IA1 and IA2, awaiting further evidence for routine use (2).

### Evaluation and size of metastases in the sentinel lymph node

Nodal involvement encompasses macrometastasis (MM; tumor deposit >2 mm), micrometastasis (MIM; tumor deposit >0.2 mm up to 2 mm), and isolated tumor cells (ITC tumor deposit up to 0.2 mm). According to TNM 8, MM is considered pN1, MIM pN1(mi), and ITC pN0 (34, 35). Studies have yet to define the prognostic impact of these distinctions.

SLN evaluation is more precise than pelvic lymphadenectomy due to ultrastaging, which increases the likelihood of detecting MIM. Multiple studies, both retrospective and prospective, demonstrate high sensitivity of SLN for detecting lymph node involvement. For instance, the prospective French SENTICOL study reported a sensitivity of 92% and a negative predictive value of 98.2% (36). Similarly, a significant retrospective study analyzing SLN sensitivity in CC reported 91% sensitivity in the entire cohort and 97% in the bilateral migration subgroup (37).

Debate surrounds the intraoperative evaluation of SLN due to its low sensitivity (53–89%) and increased tissue management complexity, potentially resulting in undetected MIM, ITC and even MM (38, 39). Nevertheless, intraoperative evaluation can detect most MM (82.1%), potentially avoiding combined treatment if surgery is abandoned, and the patient is referred for primary chemoradiation (40). Therefore, patients should be informed before surgery that despite negative intraoperative SLN evaluation, metastases may be identified later in 30–50% of cases with ultrastaging (41).

### Technical recommendations

ESGO/ESTRO/ESP guidelines endorse performing SLN with indocyanine green (ICG) as the preferred technique, another technique is combined blue dye and radiocolloid. A non-inferiority study, “FILM,” comparing ICG and isosulfan blue, demonstrated better ICG detection rates (42). ICG offers advantages over patent blue, reducing the risk of anaphylaxis, and over radiotracer, as it avoids handling radioactive materials.

For radioisotope use, specifically Technetium-99, two injection protocols exist: a long one with 120 MBq injected one day preoperatively and a short one with 60 MBq injected on the morning of the operation. It is advisable to combine lymphoscintigraphy with patent blue. For colorimetric methods, blue dye or ICG is used, with injections performed superficially and deeply once surgery has commenced, as dye migration occurs within 10 to 15 minutes (42, 43).

The initial step involves verifying lymphatic channel migration with the peritoneum closed, followed by exploration of external iliac territories, iliac bifurcation, and obturator region, ensuring complete exposure of relevant structures. Lymph nodes should not be removed without visualizing their afferent channel to avoid resecting non-sentinel nodes. Systematic searching for routes and SLN in atypical territories should be conducted (43).

No specific CC study provides information regarding the number of cases constituting the learning curve. However, some retrospective studies on endometrial cancer suggest around 30 to 40 cases (44, 45).

**Highlights:** In the **Table 1** we show the main articles about this topic.

1. Standard lymph node evaluation in early-stage CC involves bilateral pelvic lymphadenectomy.
2. SLN can be considered in patients with stage IAI CC with PLV and IA2, IB1, IB2, and IIA1.
3. Intraoperative SLN study may be considered for patients at higher risk of lymph node involvement, balancing the risk of tissue loss for detecting micrometastases and isolated tumor cells.
4. Any suspicious lymph node should undergo intraoperative evaluation.
5. Preferred techniques include ICG, with patent blue + radiocolloid considered an acceptable alternative.

**Table 1:** Sentinel node articles reviewed.

Publication year	Authors	Type of study	Objective	Results
2011	Lécuru F et al.	Prospective, multicenter	Sensitivity and NPV (negative predictive value) of Sentinel lymh node (SLN)	-Detection with blue dye and radioisotope of 97.8% (95% CI, 93.8% to 99.6%) -Sensitivity of 92.0% (23 of 25; 95% CI, 74% to 99%. -NPV 98.2% (111 of 113; 95% CI, 74% to 99%) for detection of lymph node metastases.
2012	Cibula D et al.	Multicenter cohort, retrospective	FN (false negative) rate of ultra staging of SLN in operable cervical cancer (CC) in clinical stage IA-IIB.	-FN rate of 2.8% (1.3% in bilateral detection) -Sensitivity of ultrastaging 91% and bilateral detection 97%
2013	Martínez A et al.	Retrospective cohort	Diagnostic accuracy of frozen section in SLN	-Sensitivity and NPV of macrometastsis 100% -Sensitivity for macro and micrometastasis 88.9% and NPV 98.8%
2013	Slama J et al	Retrospective cohort	Diagnostic accuracy of frozen section for SLN in clinical stage IA2-IIB	-Macrometastasis Sensitivity 81% VPN 94% -Global Sensitivity 56%(excluding isolated tumor cells (ITC) 63%) Specificity 100% VPN 0.83 (excluding ITC 0.91)
2018	Frumovitz M el al	Phase III, multicenter, randomized, non inferiority study	Compare SLN detection with Indocyanine green (ICG) vs isosulfan blue in CC	-Detection of 1 or more SLN with ICG 97% (bilateral 81%) - Detection of 1 or more SLN with blue 47% (bilateral 32%)
2020	Cibula D et al	International, multicenter prospective observational trial.	Secondary: intraoperative analysis of SLN	Macrometastasis sensitivity 75.9% Macro and micrometastasis sensitivity 45.8%
2023	Agusti N et al	Systematic review and meta-analysis	Diagnostic accuraty of frozen section in SLN vs ultrastaging	Group sensitivity 65% Sensitivity excluding ITC 72%

### Less than radical surgery

While radical hysterectomy/trachelectomy and bilateral pelvic lymphadenectomy constitute the standard treatment for cervical cancer, these procedures carry complications affecting quality of life (e.g., sexual and bladder dysfunction). Recent theories propose that low-risk patients, defined by specific tumor characteristics, could safely undergo less radical surgery, such as cone or extrafascial hysterectomy with lymph node evaluation. The ConCerv study demonstrated the feasibility of such less radical surgery in low-risk patients, with a 2-year recurrence pattern of 3.5% (9). The SHAPE study, a prospective phase III non-inferiority study comparing extrafascial hysterectomy against radical hysterectomy in low-risk patients (stage IA2 – IB1, squamous-cell carcinoma, adenocarcinoma or adenosquamous histology, tumors <2cm, with limited depth of cervical stromal invasion (less 10 mm) obtained by diagnostic loop electrosurgical excision procedure or conization or by preoperative pelvic magnetic resonance showing < 50% stromal invasion, w/not PLV); evidencing non- inferiority with a recurrence rate at 4.5 years (46), with less adverse events in extrafascial hysterectomy group however is necessary take these results with caution and know how to carefully select the patients who will benefit from this treatment.

**Highlights**

1. Low-risk cervical cancer patients may benefit from less radical surgery, such as type A/cone hysterectomy, with lymph node evaluation. However, awaiting SHAPE study publication for oncological safety confirmation is advised.

### Incidental cervical cancer diagnosis after hysterectomy

The incidence of cervical cancer discovery after hysterectomy ranges from 3.5% to 1.9%, attributable to factors like inadequate preoperative evaluation or errors in clinical or pathology evaluation, leading to suboptimal treatment. Patients without residual disease by imaging studies post-hysterectomy may benefit from complementary surgery + adjuvant treatment, demonstrating improved survival compared to those without complementary surgery, nevertheless is necessary to evaluate each case and evaluated the patient (functional status, presence of comorbidities, etc.), histology subtype and tumor characteristics to choose the best treatment. Subgroup analyses suggest observation may suffice for select patients, but this warrants further study (47, 48).

**Highlights**

1. Pathology review by an expert is essential post-hysterectomy.
2. Imaging studies should assess residual disease presence.
3. Multidisciplinary team evaluation is crucial.
4. Patients with residual disease or adverse prognostic factors should receive standard treatment.
5. Complementary surgery consideration should align with disease prognostic factors and treatment necessity.

## Conclusion

Cervical cancer treatment has evolved to become increasingly individualized, necessitating a multidisciplinary team approach considering tumor characteristics and patient preferences to determine the optimal therapeutic strategy.

## Author contributions

CN-M: Writing – original draft, Writing – review & editing. RH-R: Writing – original draft, Writing – review & editing. DI-O: Writing – original draft, Writing – review & editing. RS-H: Conceptualization, Data curation, Formal analysis, Funding acquisition, Investigation, Methodology, Project administration, Resources, Software, Supervision, Validation, Visualization, Writing – original draft, Writing – review & editing.
